# A deep learning approach for designed diffraction-based acoustic patterning in microchannels

**DOI:** 10.1038/s41598-020-65453-8

**Published:** 2020-05-26

**Authors:** Samuel J. Raymond, David J. Collins, Richard O’Rorke, Mahnoush Tayebi, Ye Ai, John Williams

**Affiliations:** 1 Dept. Civil and Environmental Engineering, Massachusetts Institute of Technology, Cambridge, MA 02139 USA; 2 Center for Computational Science and Engineering, Massachusetts Institute of Technology, Cambridge, MA 02139 USA; 3 Biomedical Engineering Department, The University of Melbourne, Melbourne, 3010 Australia; 4 Engineering Product Design Pillar, Singapore University of Technology and Design, Singapore, 487372 Singapore

**Keywords:** Biomedical engineering, Computer science, Acoustics

## Abstract

Acoustic waves can be used to accurately position cells and particles and are appropriate for this activity owing to their biocompatibility and ability to generate microscale force gradients. Such fields, however, typically take the form of only periodic one or two-dimensional grids, limiting the scope of patterning activities that can be performed. Recent work has demonstrated that the interaction between microfluidic channel walls and travelling surface acoustic waves can generate spatially variable acoustic fields, opening the possibility that the channel geometry can be used to control the pressure field that develops. In this work we utilize this approach to create novel acoustic fields. Designing the channel that results in a desired acoustic field, however, is a non-trivial task. To rapidly generate designed acoustic fields from microchannel elements we utilize a deep learning approach based on a deep neural network (DNN) that is trained on images of pre-solved acoustic fields. We use then this trained DNN to create novel microchannel architectures for designed microparticle patterning.

## Introduction

Microfluidic platforms are an important tool for precise micromanipulation, using force gradients on the length scale of cells and microparticles themselves. A major application space in microfluidics is the patterning and long-term retention of living cells; patterning is a powerful tool for the formation of cellular spheroids^1^, tissue engineering^2^, drug screening^1^ and single-cell analysis^3^, cellular interactions^4^, and mechanobiology^5^. The design of microfluidic systems for these activities, however, typically occurs on a first-principles basis and generally omits parametric optimization of the microchannel features. This has a large impact when hydrodynamic forces are involved, since the channel geometry can affect how, for example, lift and drag forces are distributed. In the case of hydrodynamic effects there has been some work using numerical simulations^6,7^ to configure channels to optimize their mixing and dilution characteristics, showing the promise of modifying channel shapes for creating optimized microfluidic devices.

Actively applied forces, rather than just hydrodynamic ones, however, are increasingly integrated into microfluidic devices for more refined and dynamic actuation. Acoustic fields are a particularly useful method for micromanipulation due to their biocompatibility, high force magnitudes arising from MPa order pressures and wavelengths that can match the length scale of individual cells. To date, acoustic fields have shown their capacity for versatile microscale actuation activities including patterning^8^, acoustic streaming^9–11^, droplet manipulation^12^, cell cultures^13,14^, mixing^15,16^ and sorting^8^, with actuation down to the single cell level^12,17^. In the case of patterning, these fields have typically been limited to creating simple lines^18^ or grids^19^ of cells and microparticles due to the limitations imposed by the transducers and channel geometries. For the most part these channel shapes are rectilinear for both resonant channels^20^ and substrate-wave^21^ based designs. To replicate the cell patterns found in human tissues or to study cellular interactions in other arrangements, however, an alternative methodology is required to create acoustic fields that can be used to guide cells in more tailored configurations.

**Figure 1:**
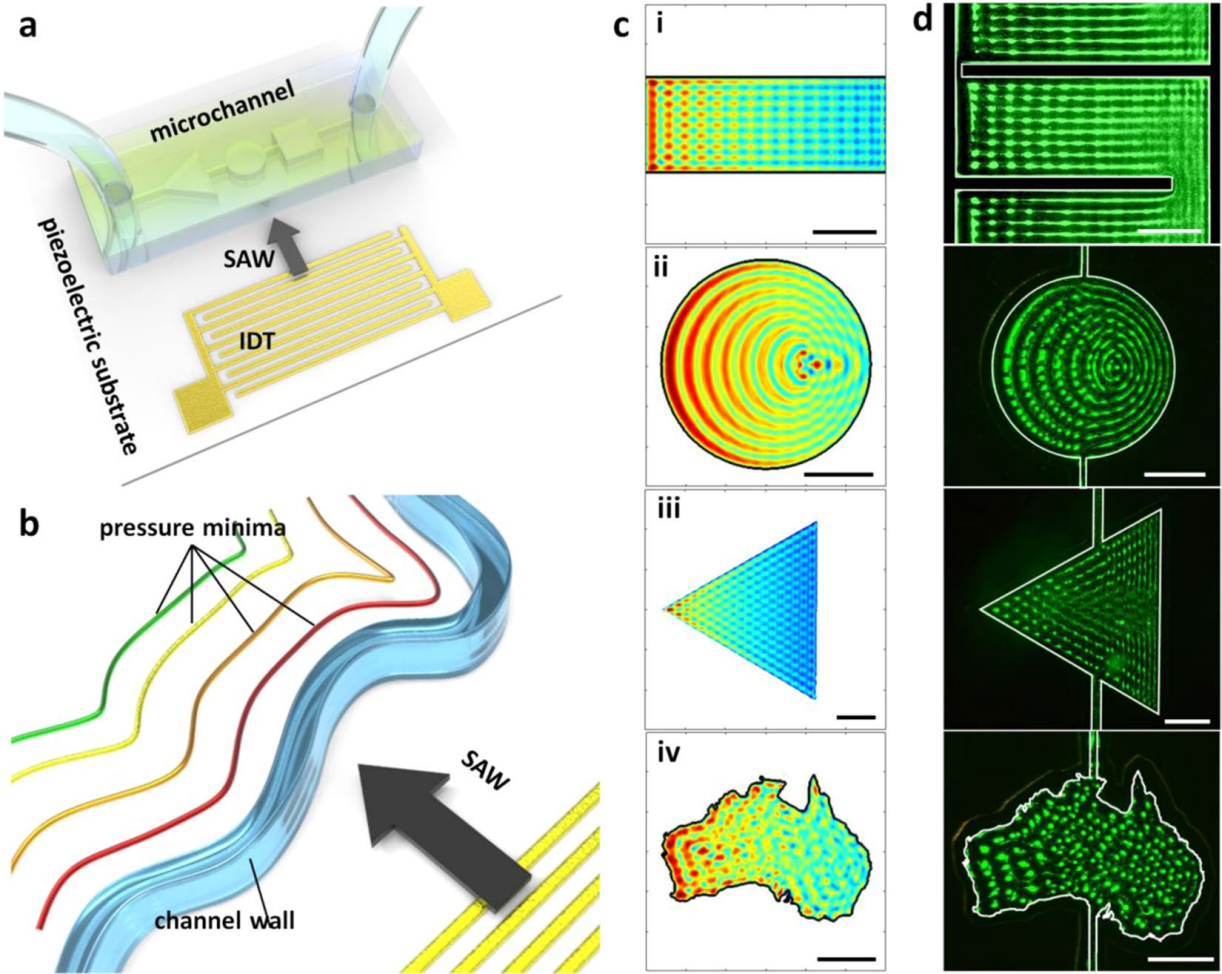
Channel-boundary induced patterning with the application of a surface acoustic wave (SAW). (**a**) A shaped microchannel in the path of a SAW yields (**b**) pressure minima in the fluid domain arising from diffractive channel interaction effects, with alignment parallel to channel walls. A SAW is generated by an alternating electrical signal across an interdigital transducer (IDT). (**c**) Altering the channel configuration results in different acoustic fields, here showing (i-iii) different simple shapes and (iv) the country of Australia (apologies to Tasmania). (**d**) The arrangement of patterned green fluorescent 1 µm diameter polystyrene particles in shaped channels corresponds to the modelled pressure minima locations.

While holographic plate metamaterial^22–24^ and micro/nanopore interfaces^25^ are examples of such approaches, a recent set of publications has demonstrated promise for creating defined acoustic fields by using microchannel walls to modify the acoustic force landscape^26,27^, with the advantage of being readily applicable to microscale, >10 MHz order acoustic fields and straightforward implementation by virtue of the actuation and fabrication methods employed. In these works, it was revealed that limiting the spatial extent of a travelling surface acoustic wave (SAW) creates a diffractive acoustic field with a defined spatial periodicity in the vicinity of channel boundaries. Since the time-averaged acoustic nodal positions that develop are parallel to the channel walls, this permits acoustic fields that can be easily modified without requiring complex integrated multi-transducer designs^28^; the transducer itself need not be altered, only the channel boundaries. Moreover, this results in time-averaged periodic force field gradients, effectively standing waves from the perspective of a suspended microparticle, with the application of only a single travelling wave. While to date only straight and simple curved lines of particles have been shown, in principle this also permits more complex non-uniform acoustic patterns. By changing the orientation of the microchannel walls relative to the incident acoustic field the acoustic field nodal lines can be angled in arbitrary configurations. In Fig. 1 we show the concept of channel-boundary induced acoustic patterning. Figure 1a shows a generalized device layout, with an interdigital transducer (IDT) generating a SAW, which creates a spatially nonuniform acoustic field in a shaped microchannel. Figure 1b shows that by using a channel wall material whose acoustic impedance is less than that of an adjoining fluid domain a condition called total internal reflection (TIF) develops^29^, where acoustic sources from behind the wall are unable to contribute to the fluid domain field, resulting in periodic pressure minima that are parallel to the channel walls. The pattern spacing (the distance between neighboring pressure minima) radiating away from a channel wall is denoted by ${\lambda }_{{\rm{\theta }}}$, which for a flat wall is given by^30^

1 $${\lambda }_{{\rm{\theta }}}={\lambda }_{l}\,\sin ({\rm{\theta }})\csc \left({\rm{\theta }}-{\sin }^{-1}\left(\frac{{c}_{f}}{{c}_{s}}\,\sin \,\theta \right)\right),$$

where ${c}_{f}$ and ${c}_{s}$ are the fluid and substrate sound speeds, ${\lambda }_{l}$ is the acoustic wavelength in the fluid and ${\rm{\theta }}$ is the orientation of the channel wall relative to the SAW propagation direction. Since channel walls can take on essentially any shape, with different values of ${\lambda }_{{\rm{\theta }}}$ for different channel wall orientations, the arrangement of the acoustic field can take on a similarly wide variety of conformations. The relevance for microfluidic patterning activities is that by changing the orientation and morphology of the channel walls, the resulting acoustic field – and therefore particle pattern – can be defined. In the case of a square channel wall, the application of a SAW (propagating from left to right) results in an acoustic field that is a superposition of the acoustic fields from each of the channel walls, each of which with a different ${\lambda }_{{\rm{\theta }}}$, thereby giving a grid of acoustic pressure minima with different acoustic periodicities in the vertical and horizonal directions. Figure 1c shows the modelled^31^ pressure field distribution in microchannels in the shape of (i) a rectangle, (ii) a circle, (iii) a triangle and (iv) Australia; we can see here that the shape of the channel boundaries has a significant impact on the resulting acoustic field, with a grid, circular, diamond or highly nonuniform arrangement, respectively, of pressure minima developing. In the case of a rectangular or triangular channel, we can readily see that the patterning that develops is a result of the intersections of periodic minima from adjoining channel walls. Figure 1d shows that the particle alignment (here with 1 µm diameter fluorescent beads) follows the distribution predicted by the pressure field in Fig. 1c, with particle aggregation in the predicted local minimum pressure locations. The particle suspension was introduced into these shaped channels via smaller 40 µm wide channel conduits, seen at the top and bottom of (ii-iv). We can see that a periodic particle pattern develops parallel to the channel walls as expected, resulting in complex nonuniform patterns with the use of a variably shaped channel as in (iv). Seeing that the channel morphology has such a stark impact on the resulting acoustic field, defining channel shapes that create these more complex fields is thus a promising pathway towards generating localized, non-uniform micropatterns. The formation of the particle pattern from Fig. 1d(iv) is shown in Supplementary Video S1 as a representative example.

For these more complex fields, however, it is not immediately clear how to make the connection between what we can control (the channel geometry) and what we wish to define (the acoustic field). For all but the most regular particle grids, where the acoustic field periodic spacing can be defined by Equation 1, there is no simplistic way to design the channel shape to generate a desired acoustic field. Using an iterated optimization algorithm has been demonstrated when acoustic wavefronts were projected through a waveguide layer towards a patterning region that was a fixed distance away^24,32^. Such an approach in the case of a microchannel on a SAW substrate as in Fig. 1, however, could yield an excessively large solution space since each wall generates a periodic field, where each set of periodic lines also interacts with those from neighboring walls. Alternatively, an algorithm that changes the channel shape incrementally through successive iterations might be feasible to create improved fields, though has the challenge of requiring many thousands of guided simulation iterations since there is essentially an infinite variety of shapes. Moreover, such an optimization approach has the potential to settle in local minima, resulting in a shape that might be far from optimal. An ideal design approach would therefore take a minimum amount of time and yield an approximation of a globally optimized solution that could be reliably applied for a range of desired acoustic fields, effectively solving the inverse problem that relates the acoustic field to the resulting channel boundary. Performing a single-step analytical process is practically infeasible since the solution space is effectively unbounded due to the periodic nature of the acoustic fields that are produced. Instead, we explore an approach based on deep learning to produce channel conformations that produce a designed acoustic field, where the solution space boundary conditions can be imposed by the training data to readily produce optimized results.

Since acoustic fields vary spatially in the plane of a microchannel, defined in 2D with local variations in acoustic pressure, it’s straightforward to parse this information as an image. Deep neural networks (DNN) have demonstrated proficiency in creating novel images from image-based training data^33^, and consist of several layers of connected artificial neurons, each programmed with a unique weight and bias term that will allow for a signal to pass through (become activated) once it has passed a particular threshold value. These networks were developed originally in the 1940’s^34^ and largely ignored for several decades by the wider community until improvements in both the algorithms and computing power meant that these networks could be created in a size and sufficiently trained so that they could be useful. The power of neural networks arises from the ensemble effect of adding these connected neurons and layers. Together these systems can optimize on a dataset to generate a transfer function that accurately parses an input signal (for example the image of an animal) to an output signal (the species name of that animal). The remarkable aspect of these deep neural networks is that, with sufficiently large datasets for training and a good optimizer, they can discover relationships between almost anything that has some logical connection. This has been used to great effect in other areas of science and engineering such as the work done by Sganga *et al*. who used Convolutional Neural Networks (CNN) to help improve lung disease diagnostics^35^ by predicting the expected images that would be detected after training on videos of other lung procedures. In addition, neural networks have been used in the field of computational mechanics for some time, especially in the place of a constitutive law of complex viscoelastic rate-dependent materials^36^ where experimental data was used to train the network to elucidate stress-strain relationships.

In our case, there is a clear connection between the acoustic boundary conditions and the resultant acoustic field and vice versa. Whereas the acoustic field can be directly calculated if the channel boundaries are known^31^, in order to create a designed acoustic field we use a DNN to infer the boundary conditions from a simulated pressure field. The result of this approach is that a desired pressure field (where we want particles to aggregate) can be defined, whereafter the DNN produces the requisite channel boundary conditions (the channel geometry used to produce the desired pressure field).

## Results

### Training data and acoustic model

A large volume of training data is required to optimize the activation function thresholds in each of the DNN layers. While datasets exist to help train specific network types (eg. those used for simple computer vision such as the Modified National Institute of Standards and Technology (MNIST)^37^, and Canadian Institute For Advanced Research (CIFAR) datasets^38^), there is no such dataset for acoustic fields. Though the selection of training data is a non-trivial task that requires consideration in its amount and variety, the advantage of the DNN approach is that once this is trained the channel geometry can be rapidly computed for a given acoustic field design. For this work, we seek to synthesize training data for acoustic fields in microfluidic devices. Generally speaking, ideal qualities of training data are that the dataset should be large enough to avoid overfitting the network, data points should be of good enough quality such that any noise within the entirety of the dataset follows a regular distribution, and that variables in the data should have a strong logical connection such that the input-output inferences can be easily learned by the network during training. In the case of mapping acoustic fields to channel geometries, this requires that the training data should comprise a wide number of different shapes with the resolution of the modelled pressure fields well below that of the acoustic wavelength. Since the acoustic field is directly determined by the channel shape, the need for a logical connection between input and output layers is satisfied. To generate this training data, we utilized a simulation approach from our previous work^31^ that produces an acoustic pressure field from a given channel geometry. This solves for the acoustic pressure field given a set of boundary conditions, namely an oscillating substrate that is bounded by a channel geometry. This simulation engine solves for the pressure field according to the Huygens-Fresnel principle, which states that the acoustic field at a given point in the fluid is equal to the linear superposition of all contributing spherically propagating wavefronts from a transducer surface. Using this engine we can take a geometry, simulate a SAW and then calculate the pressure field within the fluid. This analytical model solves in 10–30 milliseconds on a typical personal computer for the 300 × 300 pixel acoustic pressure fields that are calculated. The benefit of learning from these simulation outputs is that we essentially have limitless amounts of high-quality data.

For the acoustic-driven displacements that produce these pressure waves, we consider the surface normal component of the substrate velocity as the boundary condition (BC) for acoustic coupling into the liquid. For a traveling SAW beneath a liquid channel on 128°-YX lithium niobate, this is given by^31,39^

2 $$v(x,t)=\omega {\xi }_{0}{e}^{-\alpha x}{e}^{i\omega t}{e}^{-ikx}$$

where $\omega =2\pi f$ is the angular frequency, $k$ is the wave number, ${\xi }_{0}$ is the displacement amplitude, $x$ is the distance from the channel edge along the $x$-direction and $\alpha$ is the SAW amplitude decay coefficient^8^. We omit the time parameter, however, when calculating for the acoustic pressure field since the solution is found in the frequency domain from which a time-averaged solution is calculated. According to the Huygens-Fresnel Principle the acoustic velocity amplitude can be found through the summation of the propagating spherical wave sources that contribute to any given point. The dimensionless spherical wave function (SWF) defines the spatial periodicity of a spherical pressure wave in a liquid and is given by

3 $${\rm{SWF}}={(R+1)}^{-1}{e}^{-\frac{i\omega R}{{c}_{l}}}$$

where ${c}_{l}$ is the acoustic velocity in the liquid and $R$ is the distance from a spherical wave point source. The inverse-square relationship between the intensity and the distance means that the pressure amplitude decays linearly with $R$, which we replace with $R+1$ to normalize the SWF to unity at the substrate/liquid interface (where $R=0$). To create the final pressure field, which is the metric used to inform device design, a convolution of the BC in Eq. (2) and the SWF in Eq. (3) is required, such that the pressure, $P$, at any point is the sum of all spherical waves emanating from the boundary, with

4 $$P=[{\rm{SWF}}\ast {\rm{BC}}].$$

Since we are interested in the time-averaged pressure amplitude, this is given by

5 $$P=0.5\sqrt{(P\ast \bar{P})}$$

where $\bar{P}$ is the complex conjugate of $P$. This time-averaged pressure field can then be used to give an indication of particle settling locations in a physically realized system; while this model ignores velocity contributions to the acoustic pressure field (that arising from oscillatory fluid particle motion, and often acting contrary to the pressure field), this is appropriate in the case of most aqueous particles suspensions, such as polystyrene particles in water, where the pressure contributions are approximately an order of magnitude higher than velocity ones^40^.

**Figure 2:**
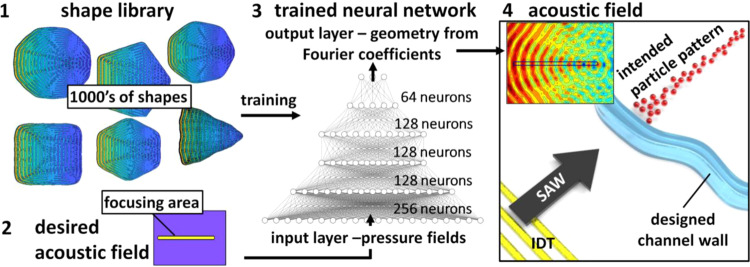
Procedure to generate channel geometries that produce designed acoustic fields. (1) A shape library, consisting of 1,000’s of individual acoustic fields (here showing a few modified polygonal shapes as examples) is used to train a deep neural network (DNN) so that the training input results in the channel geometry used to create that acoustic field. (2) We define a novel acoustic field that we feed into this (3) trained DNN. The output of the DNN is a series of Fourier coefficients that define a channel geometry. (4) The modeled acoustic field in this geometry produces the desired acoustic field, in this case a focusing region in which the intended particle pattern forms.

Figure 2 shows the overall process by which the channel geometries can be derived from input acoustic fields, with a shape library being used to train a DNN, whereafter a target acoustic field is input to derive a channel geometry that will produce that acoustic field morphology. The desired acoustic fields and shape library are input into the DNN via a vectorization process that transforms the two-dimensional geometry into a one-dimensional vector, with each row of pixels in the 2D in the field concatenated on to each other as per Supplementary Fig. S1. This geometry is represented in the form of a Fourier coefficient representation to capture the essential features of the channel shape while minimizing the number of output neurons required.

### Geometric shapes from Fourier coefficients

**Figure 3:**
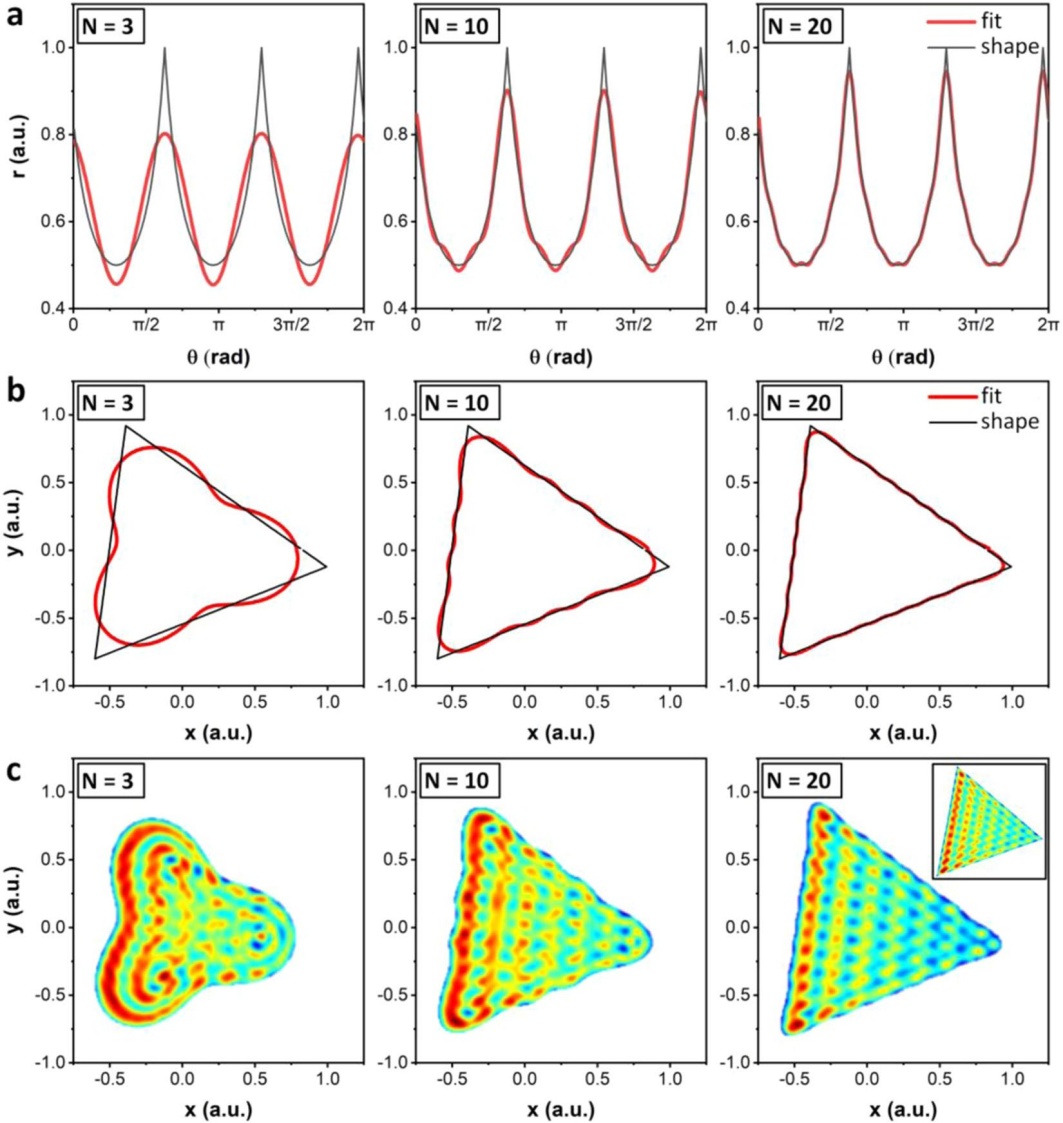
Fourier series channel geometry representations, used as the output format from the DNN. (**a**) The radius of a given shape as a function of θ can be mapped (here in black), where its Fourier series approximation can be calculated from Eq. 6 for a given value of ***N***, here for ***N*** = 3, ***N*** = 10 and ***N*** = 20. (**b**) These approximations can be superimposed on the shapes, here showing a 20° rotated equilateral triangle, demonstrating that increasing values of ***N*** provide better channel geometry fits. (**c**) The acoustic field increasingly matches that of the actual channel geometry (inset at right) for increasing values of ***N***. The SAW wavelength used here is equal to 0.14 a.u. We use ***N*** = 20 for our work since it provides a reasonable approximation of the shape and acoustic field while minimizing the required number of trained neurons in the DNN.

Having described the model that is used to generate the acoustic fields in a given channel geometry, we now require a description of the channel shape that can be readily extracted from the DNN output layer. To help limit the potential output parameter space we use a mathematical description of the channel shape that can be represented by a handful of variables. Since an arbitrary function over a given interval can be approximated by a Fourier series, a two-dimensional geometry can be created from this approximated function by using its output as a shape’s radius. The radius values given as Fourier series approximation is given by

6 $${r}_{{\rm{fit}}}({\rm{\theta }})=\frac{{A}_{0}}{2}+\mathop{\sum }\limits_{n=1}^{N}{A}_{n}\,\cos \,n{\rm{\theta }}+{B}_{n}\,\sin \,n{\rm{\theta }},$$

where ${A}_{0}$ represents the minimum radius value and ${A}_{n}$, ${B}_{n}$ are the Fourier coefficients for a given $n$, θ is the value of theta between 0 to 2π and N is the coefficient integer. Since the Fourier series is periodic every $2\pi$, discontinuities are avoided between adjoining discretized values of θ. This means that at the point where the first (${\rm{\theta }}$ = 0) and last point (${\rm{\theta }}$ = 2π) meet, there are no jumps or sharp edges, this enables straightforward translation of shapes to the simulation domain and for straightforward fabrication. The value of these ${A}_{n}$, *B*_*n*_ coefficients can be directly calculated (see elsewhere^41^). Figure 3 shows how the Fourier coefficients in Eq. 6 can be used to define a two-dimensional shape. Taking an example case where we wish to develop coefficients that approximate a 20° rotated triangle shape, we first plot the shape in terms of its radius as a function of ${\rm{\theta }}$ (black line in Fig. 3a), then calculate the Fourier coefficients and the approximated radius, here for coefficient integer values of *N* = 3, *N* = 10 and *N* = 20 (red lines in Fig. 3a). The coefficients for the *N* = 3 case, for example, are ${A}_{[1,2,3]}\,=$ [–0.118, 0.189, 19.166] and ${B}_{[1,2,3]}\,=$ [0.019, –0.067, –11.794], with the total number of coefficients (including ${A}_{0}$) equal to $2N+1$. Plotting these approximated shapes in the $x$-$y$ plane simply requires the coordinate transformations ${x}_{{\rm{fit}}}={r}_{{\rm{fit}}}\,\cos \,{\rm{\theta }}$ and ${y}_{{\rm{fit}}}={r}_{{\rm{fit}}}\,\sin \,{\rm{\theta }}$; the Fourier-series approximation is then plotted by connecting $({x}_{{\rm{fit}}},{y}_{{\rm{fit}}})$ points for adjoining ${\rm{\theta }}$ values, as shown in Fig. 3b. We can see here that the value of *N* has a strong impact on the degree to which the Fourier-series shape approximates the original one, with increasing *N* resulting in improved approximations through the incorporation of coefficients representing higher-frequency (and thus higher spatial resolution) contributions. Most important for acoustic field design, however, is whether the coefficient-defined shape’s resolution is sufficient to capture the range of acoustic fields that can be produced. Figure 3c shows the modelled acoustic pressure field inside the shapes produced with different *N*-values, where increasing the number of coefficients improves the resemblance of the acoustic field to that produced in the original shape (Fig. 3 right inset). A simple test of whether the number of coefficients is enough for the purpose of training our DNN is whether the acoustic field produced from that approximation can capture the essential features of the acoustic field from a rectilinear polygon such as the triangle used here. A value of *N* = 3 is clearly unable to represent flat channel walls, and because the periodic spacing is a function of channel wall orientation as per Equation 1, this coefficient index value produces a very poor approximation of the acoustic field. Increasing this to *N* = 10 improves this approximation, creating a mostly flat interface along the edges and thus recovering the acoustic periodicity in the SAW propagation direction, though because radius of curvature at the vertices is on the order of the SAW wavelength, the lateral periodic features (in the $y$-direction) are not fully recovered. Finally, increasing *N* to a value of 20 permits the essential features of the acoustic field from the polygonal shape to be recovered, with an identical number and locations of discernible acoustic field minima. While sharper corners are possible with further increases in *N*, this does not significantly impact the acoustic field morphology. We choose a value of *N* = 20 for the output layer of our DNN because it is a qualitative balance between achieving sufficient resolution to produce usable acoustic fields. Supplementary Fig. S2 shows that *N* = 20 is sufficient to capture the essential features for a range of polygons compared to higher *N* values, and Supplementary Fig. S3 shows that the 2D autocorrelation function residual is less than 1% with *N* = 20 for a variety of polygons. Increasing the number of coefficients beyond this further increases the possible range of coefficient permutations and the amount of training data that needs to be generated to make use of higher-order coefficients. In choosing the N value for our simulations, we recognize that there is a trade-off between the shape fidelity that can be rendered with larger N-values and the complexity, training time and required size of training dataset to accurately determine the relationship between the channel geometry and resultant acoustic field. Whereas Fig. S3 shows that the residual between *N* = 300 polygons converges to values between 10^−3^ and 10^−4^ for values of *N* ≳ 80, it is not convergence that we seek but rather the recapitulation of the distinct features of the acoustic field. The *N* value used should then be sufficiently large so that this is the case, but otherwise as small as possible in order to minimize the computational expense and training data required, as higher-order coefficient values beyond a certain *N* will not impact the acoustic field in a meaningful way. We note that the Fourier series representation can be related to spatial lengths, where higher-order coefficients correspond to higher-frequency and thus higher wavenumber sinusoidal functions. Since channel features much smaller than the acoustic wavelength do not impact the acoustic field in a directional way (i.e. Rayleigh scattering rather than Mie scattering), to this end the wavenumber (radians/distance, equal to 2π/λ) of the highest *N*-value should be significantly smaller than that of the applied acoustic wavelength. In the case of Fig. 3 where the angular wavenumber of *N* = 1 corresponds to 1 a.u.^−1^, and with a SAW acoustic wavelength of 7.1 a.u.^−1^, *N* = [3,10,20] corresponds to wavenumbers of 3 a.u.^−1^, 10 a.u.^−1^ and 20 a.u.^−1^, respectively. We can see that whereas the *N* = 10 case results in a maximum wavenumber very similar to the SAW wavenumber, and thus while it recapitulates the overall polygon shape roughly, it does not capture the nodal point distribution since a wavelength-scale feature (in this case, a radius or curvature at a corner) will produce noticeable acoustic field variations. While the specific choice of *N* is ultimately a qualitative one, based on the computational resources available in a desktop computer, a choice of N = 20 results in sufficiently small resolution features that the essential features of acoustic fields arising from even sharp-angles can be replicated, with acoustic field residuals between 10^–2^ and 10^–3^ (Supplementary Fig. S3). Using the simulation engine described earlier, a database of over 20,000 unique shapes was created by varying each of the coefficients. The pressure fields produced by these shapes were simulated using the MathWorks MATLAB R2018b platform and stored as 2-dimensional arrays of 64-bit floating point numbers.

### DNN training process

In DNN training, the weights and biases of the neurons, and the other variables, such as the kernels in the convolution layers, are adjusted so that the DNN produces minimized error. This is performed by use of a workflow known as backpropagation^42^. This error typically comes from the mean squared value of the output layer. The network is given an input, in this case the 2-dimensional pressure field array, whereafter its output is compared to the output that should have been produced. The error value is calculated and used to inform the optimizer on how to adjust the DNN variables. This process is iterated until the error value is minimized below a threshold value. We define our input as the acoustic field in the center of the 300 × 300 pixel area across which the shapes are delineated; we selected a subset of the entire modelled domain as our design region (the 151×151-point field in the center), where the acoustic field within this region is defined by the channel shape outside of it. This choice ensures that the network only ever learns the interior of the devices and so is not influenced by the boundary shapes and edge effects of the device. Since the boundary is the unknown that is being solved for we choose a constant and defined region over which the field is evaluated, where the boundary shape defines the acoustic field within this domain. We used the Neural Network Toolkit in MATLAB as this has excellent documentation and figure utilities, enabling ready integration with our simulation engine which is also coded in MATLAB. To train our neural net, an acoustic field in the design region is fed into the DNN input layer for a shape that is pre-defined by a known set of Fourier coefficients. The weight and bias values of the DNN neurons determines each of the 41 coefficient values (from *N* = 20) at the output layer, which defines the shape the DNN predicts will produce the acoustic field that was given at the input layer. The acoustic field produced by the output shape is computed using our simulation engine and compared to that of the input acoustic field. The weights and biases within the neurons are then altered according to the training algorithm, where repeating this process is used to optimize the DNN successive iterations. By using a large training data set, the DNN is essentially trained to create an internal physics model within the neuron layers that relates an acoustic field to the channel shape that is required to produce it. The training data was produced by multiplying a vector of random numbers by the Fourier coefficients with *N* = 20 for randomly chosen shapes with 3–8 sides (the latter seen in Supplementary Fig. S1), with this process performed 20,000 times. As validation of the DNN’s self-consistency, Supplementary Fig. S4 shows the outputs of the trained DNN, where the acoustic field from the design region of a novel shape produced by this process (but not included in the original training data) is extracted and used as the DNN input, where the DNN output is an essentially identical shape to that used in the input, and thus producing an almost identical acoustic field.

### DNN output and experimental validation

**Figure 4:**
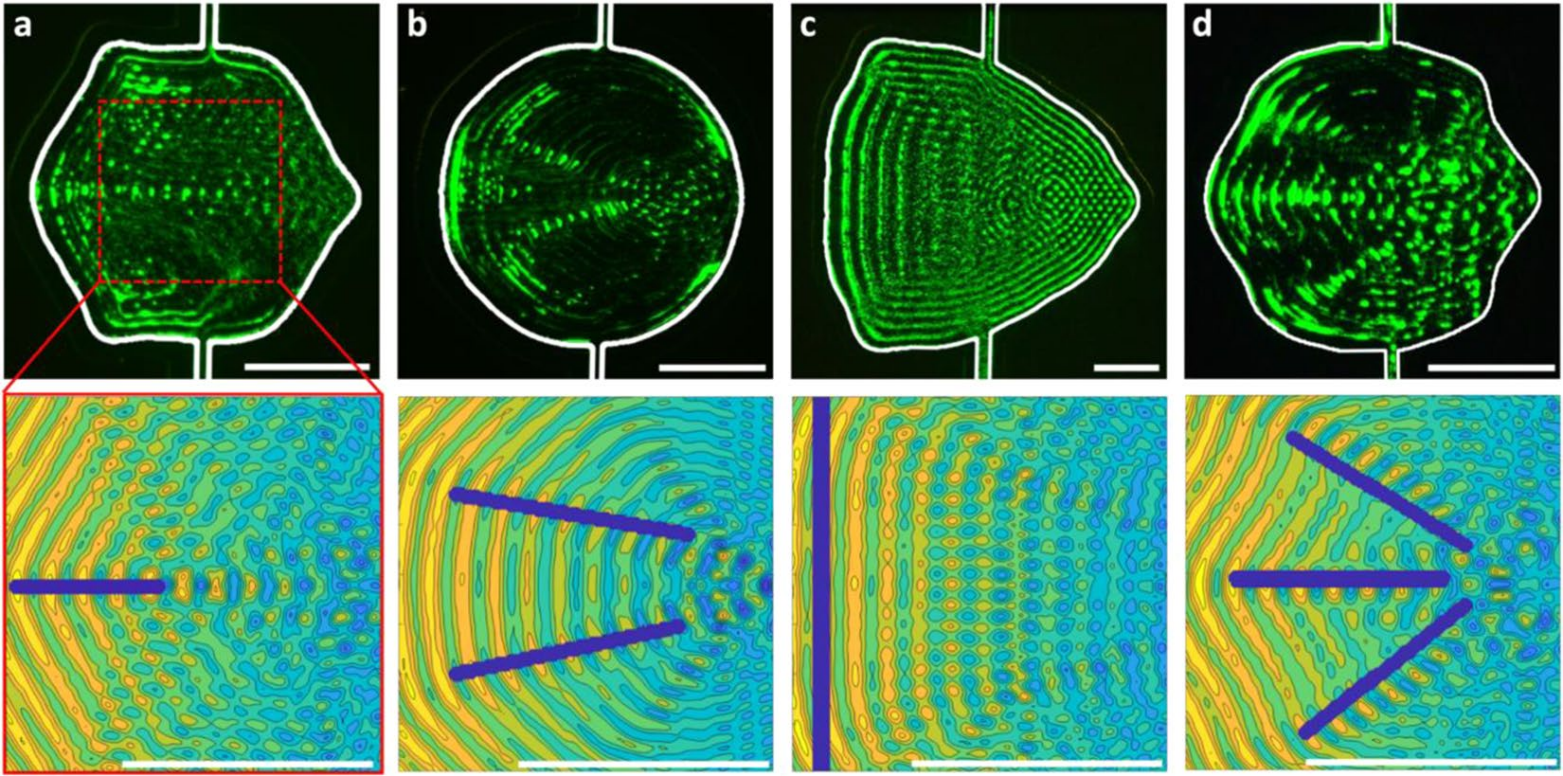
Implementation of designed channel shapes. Top image row shows patterns of suspended 1 µm polystyrene particles in different channel shapes (interior boundary highlighted in white). The applied surface acoustic wave (SAW) propagates from left to right. The bottom row shows the modeled acoustic field within the design area, 300 × 300 µm for (**a,b,d**) and 600 × 600 µm (**c**). These shapes are designed to produce (**a**) a single focusing region, (**b**) two focusing regions, (**c**) a uniform line of particles and (**d**) three focusing regions. Scale bars are 200 µm.

Having developed a trained DNN that is able to map the connections between microchannel geometry and the resulting acoustic field, we now utilize this DNN to generate channel shapes that can produce a designed acoustic field. Creating the acoustic design – where we desire the acoustic minima to be located, and thus the particle patterns to be produced – is not necessarily straightforward, since there is an inherent periodicity along the propagation direction that will change as a function of the adjoining channel wall orientation. Rather than laboriously calculate the position of each and every minima within the 2D field *a priori* with this periodicity in mind, our DNN also permits us to input approximate minima locations, namely regions defining in a general sense paths or regions where we wish particles to aggregate. This process is conceptualized in Fig. 2, showing how a target acoustic field is fed into the DNN, where in this case minima are aligned along a line in the center. Figure 4 shows such regions (in purple) superimposed on the acoustic fields that the DNN produced using these regions as the DNN input, which in essence tells the DNN that the acoustic field should contain local minima in those defined regions. While periodicity is an inherent feature of time-averaged acoustic fields with a spatially limited transducer, the overall morphology of this acoustic field can nevertheless be controlled by changing the microchannel geometry. So, while the entire modelled acoustic pressure field isn’t minimized at all points along the defined patterning region in Fig. 4a, for example, this region nevertheless contains local minima along its length. The experimental image in Fig. 4a bears this out, where 1 µm polystyrene particles aggregate along these pressure field minima in the channel center, essentially creating a focusing region in the channel center simply by changing the channel geometry. While other local minima are also visible within the wider modelled field, particles are most likely to be retained in the minima surrounded by the highest acoustic field gradients, since the acoustic force scales with the acoustic force potential difference rather than its absolute magnitude;^43^ the DNN accordingly produces an acoustic field where these gradients are highest along the defined focusing region. Within the design region (red dashed box) in the experimental Fig. 4a, for example, particles are observed to aggregate mostly only along the defined focusing region in the channel center. While other local minima outside of the designed focusing line exist, and thus in theory are capable of particle capture, particles in the vicinity of these minima are more susceptible to perturbations arising from Brownian motion, externally driven flow and acoustic streaming because the acoustic force acting to retain these particles is not as high as that along the focusing line. While particle capture outside of the boxed design region is also observed, this is in a portion of the channel that was not selected for by the DNN; because the DNN selects a shape as a function of the target acoustic field defined in the central square design region, and the optimized channel shapes are themselves not squares, the acoustic field between this area and the channel walls is by definition unconstrained.

The modelled acoustic fields and experimental results are also shown for other target acoustic fields, with two focusing regions, a single vertical line and three focusing regions in Fig. 4[b–d], respectively. In each of these the overall morphology of the modelled acoustic field is recovered in the experimental image. In the case of Fig. 4b and Fig. 4d, particles are preferentially captured in the acoustic minima within each target region (the blue lines in the model images), as was similarly the case in Fig. 4a. Figure 4c shows an example case, where rather than the target focusing region being defined over several periodic acoustic minima, the target region is aligned entirely perpendicular to the SAW propagation direction along a single minima. The DNN has thus selected for a shape that minimizes the acoustic pressure along the length of a single line. Since the acoustic field is parallel to the channel walls, this means that the channel wall is mostly flat and vertical along its left edge. Notably, the DNN was largely able to remove the lateral periodicity within this target field by producing a curved interface (aside from the flat wall at the left) that canceled the diffractive contributions from the opposing channel walls at the top and bottom. Highlighting the difficulty in doing this were the channel to be designed manually, both Fig. 4c (a triangle) and Supplementary Fig. S4 (a square) show the strong lateral periodicity that typically eventuates due to having flat channel walls other than the one that produces the periodicity along the SAW propagation direction. Interestingly, curving the channel walls in exactly the manner the DNN produced can nullify this periodicity within the design region. The periodicity in the immediate vicinity of the upper and lower channel walls (outside of the design region) is nevertheless unavoidable, and can be seen via the particle lines at the channel edges in the experimental image of Fig. 4c.

## Discussion

We have produced a deep learning approach based on a DNN that is able to relate the channel geometry to the acoustic field that develops within it. Whereas it is straightforward to calculate the acoustic field from a given geometry, and can be performed via numerical^39,44,45^, analytical^46^ direct calculation^26,31^ approaches, the inverse of this procedure is difficult to perform, especially due to the large solution space given the inherent periodicity of acoustic fields. Nevertheless, because the channel geometry and the acoustic field can be directly linked, with the latter being driven by the former, this is still a tractable problem. While acoustic fields are commonly generated via channel resonance conditions^47^, this is not ideal for creating designed acoustic fields since this imposes the constraint that the channel dimensions should be an integer value of the acoustic half-wavelength. Intersecting standing wave substrate waves^19,48^ can also be used to create acoustic patterning, though the acoustic field that develops is decoupled from the channel shape. In this work we instead utilize the new and developing technique of diffraction-based patterning, in which the imposition of a channel boundary on a SAW device results in a spatially limited transducer domain whereby periodic diffractive lobes develop according to the Huygens-Fresnel diffraction model^26,27^. The channel material in this case only needs to be a material that has an acoustic impedance mismatch with water; if the sound speed of the channel wall is less than that of the fluid, then total internal reflection prevents acoustic energy from outside the channel entering the fluid domain, whereas if it is greater than at least a portion of the acoustic energy is reflected at the wall/fluid interface. In this latter case, acoustic propagations from outside the channel will in any case be minimized by using a very thin channel wall that couples a minimum of total energy from the substrate. This channel-based patterning approach is unique and beneficial in comparison to other methods in that periodic acoustic minima can be produced with only a single travelling substrate wave, and where the locations of the acoustic field minima are directly coupled to the channel wall positions. By using multiple neighboring channel walls these periodic fields can interact with each other to produce an acoustic field that is the sum of these periodicities, where the rectangular channel shape in Fig. 1c results in 2D grid patterning due to acoustic minima that are parallel to channel walls at left/right and top/bottom. Using continuously curved (i.e. Figure 1c–ii) or arbitrarily shaped (i.e. Figure 1c–iv) channel walls, for example, these periodic fields can be fashioned into a much wider variety of acoustic fields that could not be generated via traditional SAW or channel resonance methods, and opens the possibility of creating a designed acoustic field by manipulating the channel boundaries. We use here a deep neural network (DNN) to solve this inverse problem due to a DNN’s ability to relate causally connected parameters. The DNN training and computational efficiency is facilitated via a mathematical description of the channel shape, which permits a relatively small number of output neurons compared to the DNN input. We made use of a Fourier coefficient description of channel geometry to represent channel shapes, where radius values as a function of theta can be ‘wrapped’ around a central point, and where increasing the length of the Fourier coefficient vectors increases the fidelity with which shapes can be represented; sharper corners, for example, can be represented by the higher frequency components that higher-order coefficients indicate. Though we limited the coefficient integer to 20, the DNN-designed channel geometries in Fig. 4 do not contain sharp corners or vertices that approach the limits of this coefficient number, as can be seen in Fig. 3, signifying that increasing the coefficient integer value further would not substantively impact the resulting DNN designed geometry.

A characteristic of DNNs is that the output is only as good as the input used to train it. To build a DNN that could flexibly design for a wide variety of desired acoustic field inputs, we built a catalogue of training data based on 20,000 randomized alterations of polygons. There are, however, an infinite variety of permutations of Fourier coefficients and thus channel shapes that can be produced, so that it is inevitable that a DNN’s output does not represent the absolute global optimum. Nevertheless, the training data we used here was sufficient to produce acoustic fields (via DNN-formed geometries) that provided a good match for the desired inputs. Moreover, expanding the range of training data via alternative geometry generation approaches could improve the range of shapes that our DNN could generate. By coupling this deep learning approach with a novel method to generate acoustic fields in microfluidic systems, we can create particle patterns besides the simple lines and grids that are far more typically produced. This approach has wide potential for application in micromanipulation, where precise microscale control of particles and cells has utility in microfluidic devices for diagnostics and biological studies.

While our training dataset comprised of concave shapes/polygons, one benefit of the network is that so long as the input-output relationships are maintained, we can, in theory, use any training data that fits our needs. A limitation of this workflow though, is the specific simulation model and physics being modeled, where rapid simulation is at present limited to the acoustic pressure field, without taking into account channel reflections (albeit limited with a 4% reflection coefficient at the PDMS/water boundary^49^) and acoustic streaming, both of which would require more time consuming approaches (i.e. finite element model). In addition, the type of transducer modeled (travelling wave SAW) is only one of many actuation modes that would create larger solution spaces. While this somewhat limits the range potentially useful that we can explore, where acoustic streaming in particular is useful for a range of microscale activities^50^, this is not due to a general limitation of the combination of simulation and DNNs. More exotic neural network architectures are potential areas of further investigation, including additional convolutional layers to read in complex images for targeted particle positioning and potentially to simulate acoustic streaming fields directly from acoustic pressure field distributions.

## Materials and Methods

### Defining the network

For this network we chose a fully connected, feedforward network with 4 hidden layers, the structure of which is shown in Fig. 2. While the design of network architecture is not a hard science, this configuration was found to give the best balance of accuracy, versatility, and training speed. The input layer consists of 22,801 nodes (for the 151 × 151 pixel pressure field) and the output consists of 41 output nodes, one for each of the Fourier coefficients used to describe the boundary. This way the network works as a regression tool to estimate the shape of the boundary rather than classify the shape. This allows the network to create outputs that may be partially of one shape or another, interpolating between many shape definitions. For the layer activation functions several different classes were tested (Rectified Linear Unit, ReLU), Sigmoid, Tanh, etc.), with the ReLU activation function^51^ utilized as this provided the fastest optimization times.

### DNN training

As described in Section 2, training a neural network consists of solving some optimization problem where the weights and biases of the individual neurons are adjusted to minimize a particular error function of the target (output) data. For this model we used the Adaptive Moment or ‘ADAM’ technique for the backpropagation optimization algorithm. For test and for validation, 10%, respectively of the data was used, with the remaining 80% used to train the network. All of the data was split randomly. The training was conducted in MATLAB R2018b on a workstation running Windows 10 with an Nvidia Titan RTX GPU card. The root-mean squared error (RMSE) was used to track the improvements of the network to measure the distance between the vector of Fourier coefficients predicted and the target coefficients. The training was completed after 250 epochs (Supplementary Fig. S6). For the input format, we first normalized the pressures such that any values higher than 75% of the maximum pressure were set to 1.0 and anything below this was set to 0.0. Examples of such fields are shown in the second column of Fig. S4. We chose a 75% threshold value rather than a 50% one, for example, as this reflects the nature of the design input, which are regions in which we wish the acoustic maxima to be located. Since regions of maximum constructive interference correspond to neighboring maximum values of destructive interference separated by a distance of λ_θ_/2, both local acoustic pressure maxima and minima will occur in vicinity of one another; this can be seen later in the experimental results, where even though we design for acoustic maxima along defined regions, particles aggregate in the minima that also occur there. It is thus important that the largest values, here defined as those exceeding a 75% threshold, are within the design regions to affect the desired particle patterning. Since the purpose of our DNN approach is to create distinct patterns in the field along a small number of trajectories, a 50% threshold instead might result in a DNN field output whose pressure field meets this lest strict criteria in the design regions but whose primary features (those with the highest acoustic force potentials) lie outside of them, resulting in particle patterning not matching that intended.

### DNN Verification

While minimization of the error/cost function is a necessary requirement for neural network training, the model requires further testing to validate it. Once the network was trained, it was tested via the following process: (1) Create a known geometry and simulate this with the simulation engine, (2) Use the simulated pressure and use this as an input to the trained neural network, (3) The output of a neural network will be the geometry it estimates to be needed to produce the pressure field. Use this geometry as a new input into the simulator, and (4) Compare the two geometries and pressure fields.

These steps were performed over a number of different use cases that were aimed to test out different types of patterns that the training data was intended to teach the network. Example cases are shown in Supplementary Fig. S4, with the forward simulated results on the left and the results from the neural network derived shapes on the right along with the error between the two fields, which is shown to be small and indicates that the network can reproduce the correct shapes the yield a given field. While there are some differences in the two pressure fields, the shapes are remarkably close and demonstrate that this DNN is able to make the connection between the pressure field distribution and the required microchannel boundary shape.

### Device fabrication

SAW devices are comprised of a SAW transducer and a microfluidic channel. The SAW transducer is fabricated by patterning conductive electrodes on a piezoelectric 128° Y-cut lithium niobate substrate. Electrodes are comprised of a 10 nm chromium adhesion layer under a 200 nm conductive aluminium layer, with 300 nm of SiO_2_ over the entire device surface used to prevent electrode corrosion and mechanical abrasion. The electrode patterning was produced by common microfabrication processes. Briefly, this included photolithography to create regions on the substrate that were either exposed or covered with photoresist. By sputtering metals over the entire surface, etching this resist results in metal deposition in only selected regions. Our resulting interdigital transducers (IDTs), represented in the concept image in Fig. 1a by electrodes patterned on a piezoelectric substrate, have a characteristic acoustic wavelength of either 40 µm or 80 µm and were actuated by a sinusoidal electrical signal with frequencies of 96 MHz and 48 MHz, respectively. The applied power in all cases was less than 40 mW. The microchannel was formed via conventional polydimethylsiloxane (PDMS) soft-lithography, where a photopatterned mold of SU-8 was used to create channel features, which are 25 µm high. To minimize acoustic attenuation prior to the fluid domain an air pocket is used over the top of the IDTs and along the SAW propagation path prior to the microchannel, with a 50 µm wall separating the air domain from the fluid one.

## Supplementary information

Supplementary Figures.

Supplementary Video S1.
