# Supplementary material for: A deep learning approach for designed diffraction-based acoustic patterning in microchannels
Source: Sci Rep. 2020 May 26;10:8745. doi: 10.1038/s41598-020-65453-8 (PMC7251103; doi:10.1038/s41598-020-65453-8)
Supplement: Supplementary file 1 — Supplementary Figures. [file 41598_2020_65453_MOESM1_ESM.pdf]

A deep learning approach for designed diffraction-based acoustic patterning in microchannels

Samuel J. Raymond[1,2†], *David J. Collins*[3†*], *Richard O'Rorke*[4], *Mahnoush Tayebi*[4], *Ye Ai*[4] *and John Williams*[1,2*]

[1]*Dept. Civil and Environmental Engineering, Massachusetts Institute of Technology, Cambridge, MA 02139, USA*

[2]Center for Computational Science and *Engineering, Massachusetts Institute of Technology, Cambridge, MA 02139, USA*

[3]*Biomedical Engineering Department, The University of Melbourne, Melbourne 3010, Australia*

[4]*Engineering Product Design Pillar, Singapore University of Technology and Design, Singapore 487372, Singapore*

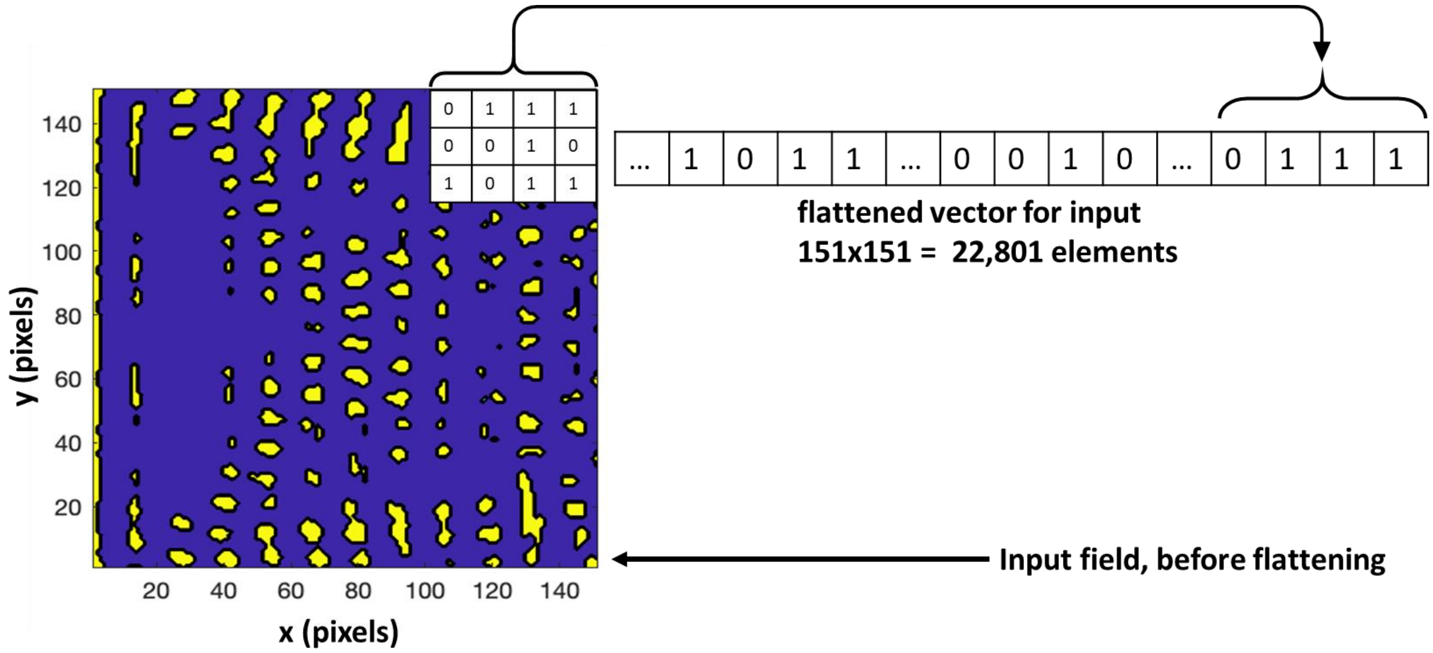

Figure S1: Representation of the flattening process used to convert the input field to the format expected by the neural network input layer. The pressures have been normalized and each row of the 2D matrix of values is flattened to a 1D vector for input into the Deep Neural Network input layer. The pressure field image shown here represents the central 150x150 pixel region of a thresholded example pressure field used as training data.

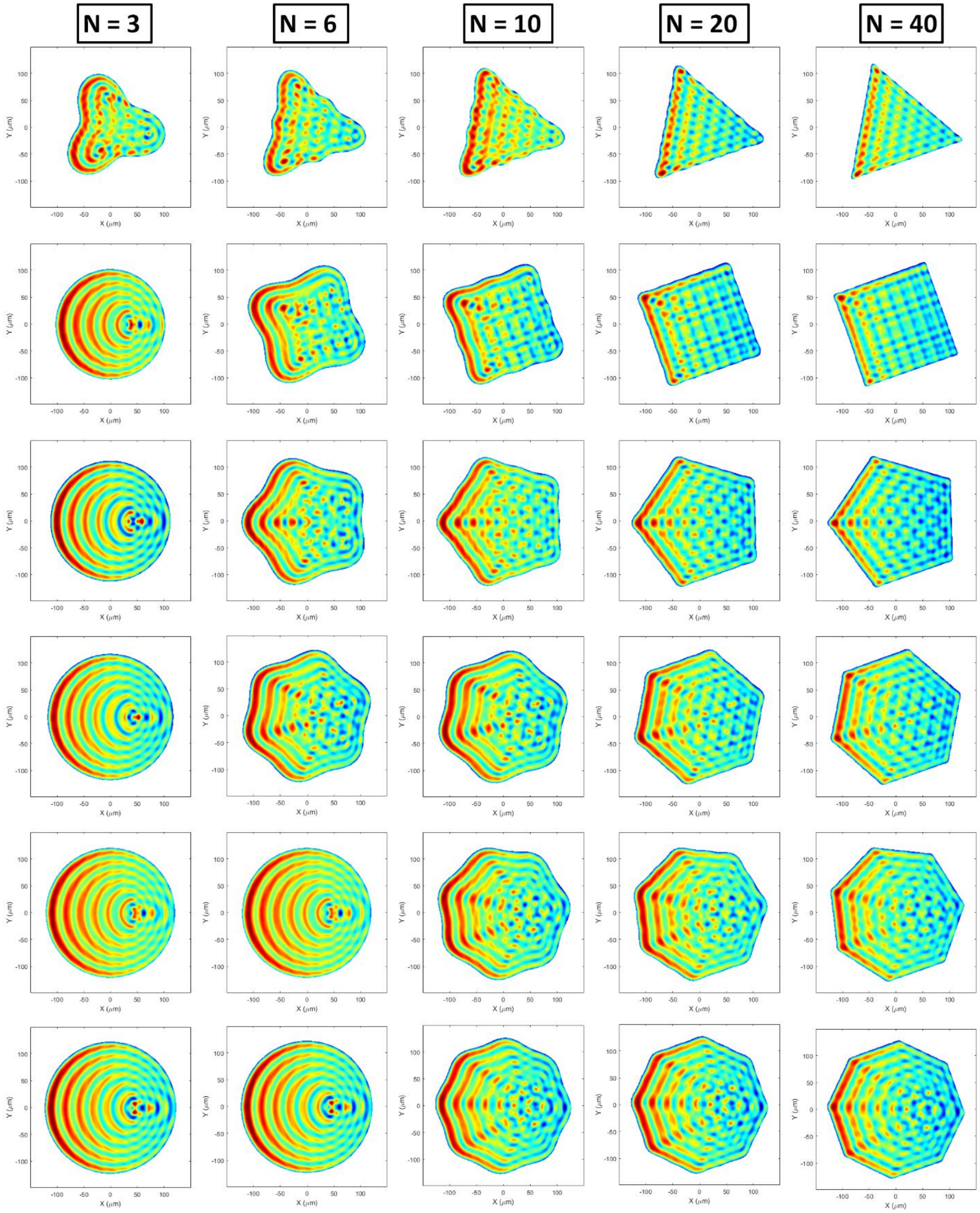

Figure S2: Modelled acoustic fields for values of Fourier coefficients indices and number of polygon vertices. Left to right shows the acoustic fields for $N = 3$, $N = 6$, $N = 10$, $N = 20$ and $N = 40$. Moving down through the rows increases the vertex number by one, with a triangle, square, pentagon, hexagon, heptagon and octagon used as the target shape from top to bottom. Notably, the Fourier coefficient index N must be greater than the vertex number for anything but a circle approximation to be produced.

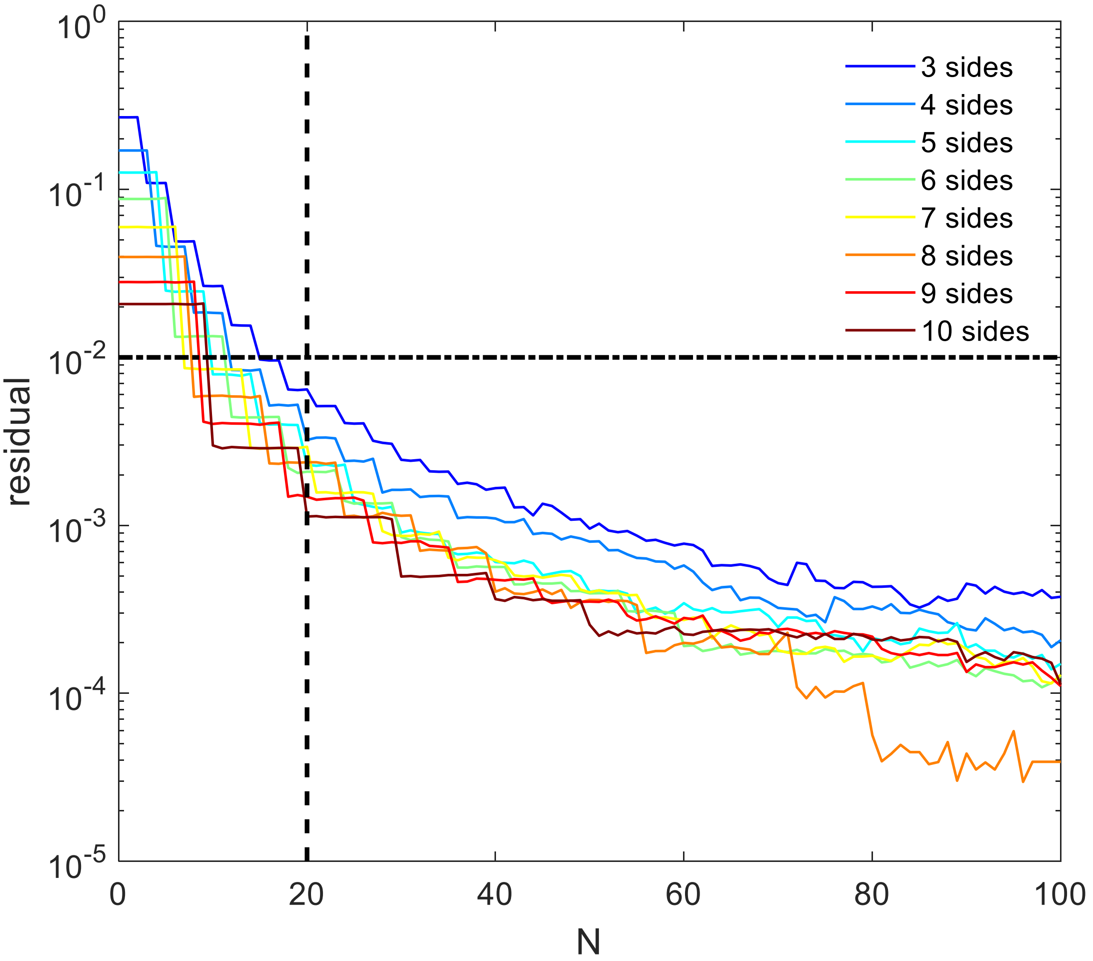

Figure S3: Effect of Fourier coefficient index N values on the ability to approximate acoustic fields. The residual here is given by one minus the normalized maximum output from the two-dimensional autocorrelation function of the acoustic field (at the given value of N) with the acoustic field from an arbitrarily high N value ($N = 300$) representing an idealized polygon. This is calculated for polygons with 3 to 10 sides. Increasing N values reduces the difference in the acoustic field produced from the idealized shape and the approximated one. The residual tends to be smaller for shapes with a larger number of sides for a given N value since these shapes tend to be better approximations of circles (see Figure S1), which can be produced with a smaller number of Fourier coefficients. An N value that reduces this residual to less than 1% (dot-dash line) for all the polygons tested is $N = 20$ (dashed line).

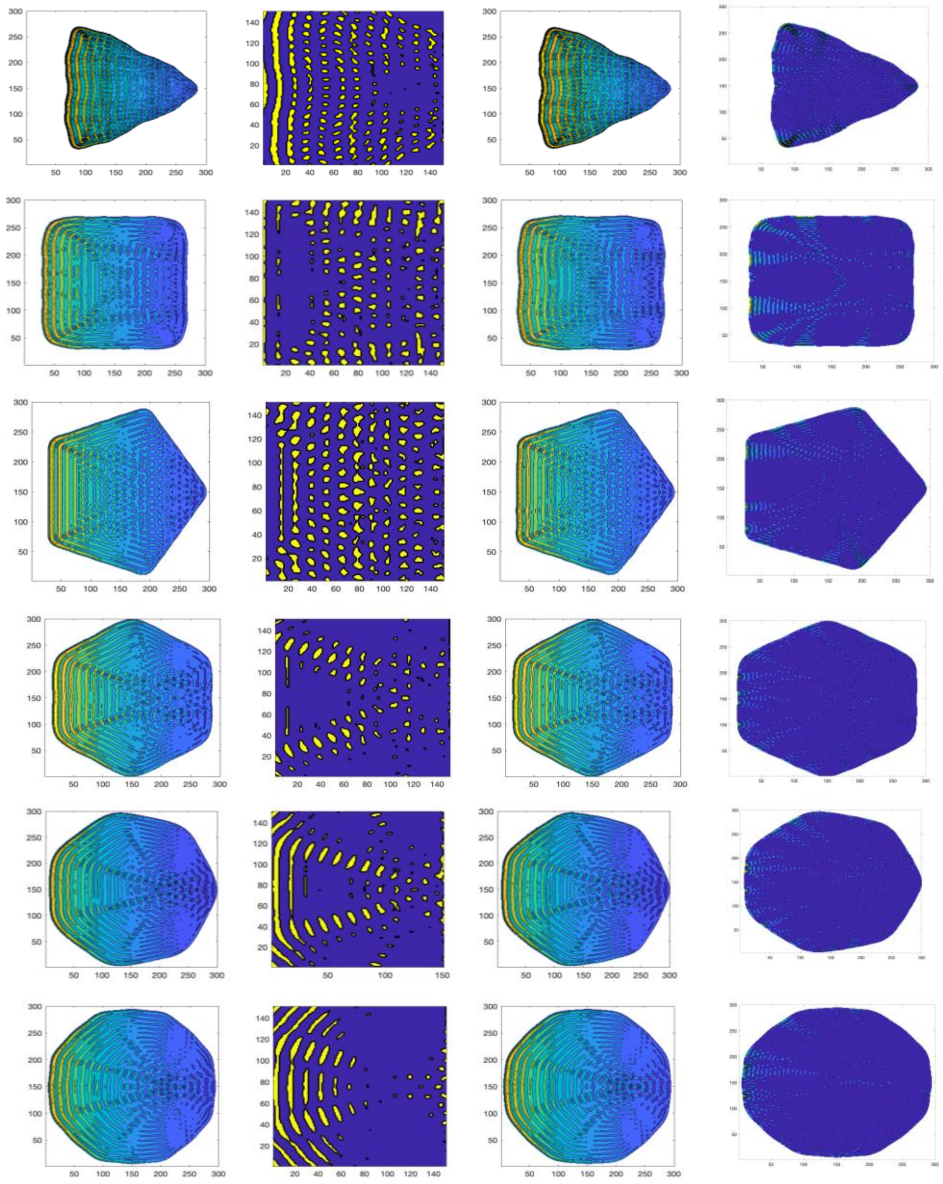

Figure S4: Validation of the neural network’s self-consistency. Here we can see how well it performs when a known shape (left images) is taken, the pressure field is then cropped and normalized (middle images) and used to generate a boundary shape from the neural network. This boundary is then used as an input to the simulator and the shape and the field are produced to compare (middle-right images). The error between the known shape input and the network-derived shapes in terms of their simulated pressure fields is shown in the far right column.

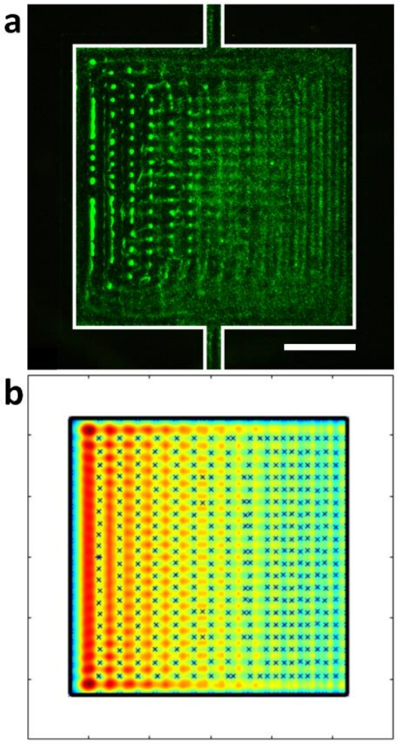

Figure S5: Particle patterning in a rectilinear (square) channel. (a) Experimental patterning of 1 µm polystyrene particles in an 800x800 µm square channel. Scale bar is 200 µm. (b) The pressure field minima (black crosses) are superimposed on the modelled field, showing that periodic patterning develops not just in the SAW propagation direction (left to right) but also laterally. This can be compared to the designed shape from Figure 4c.

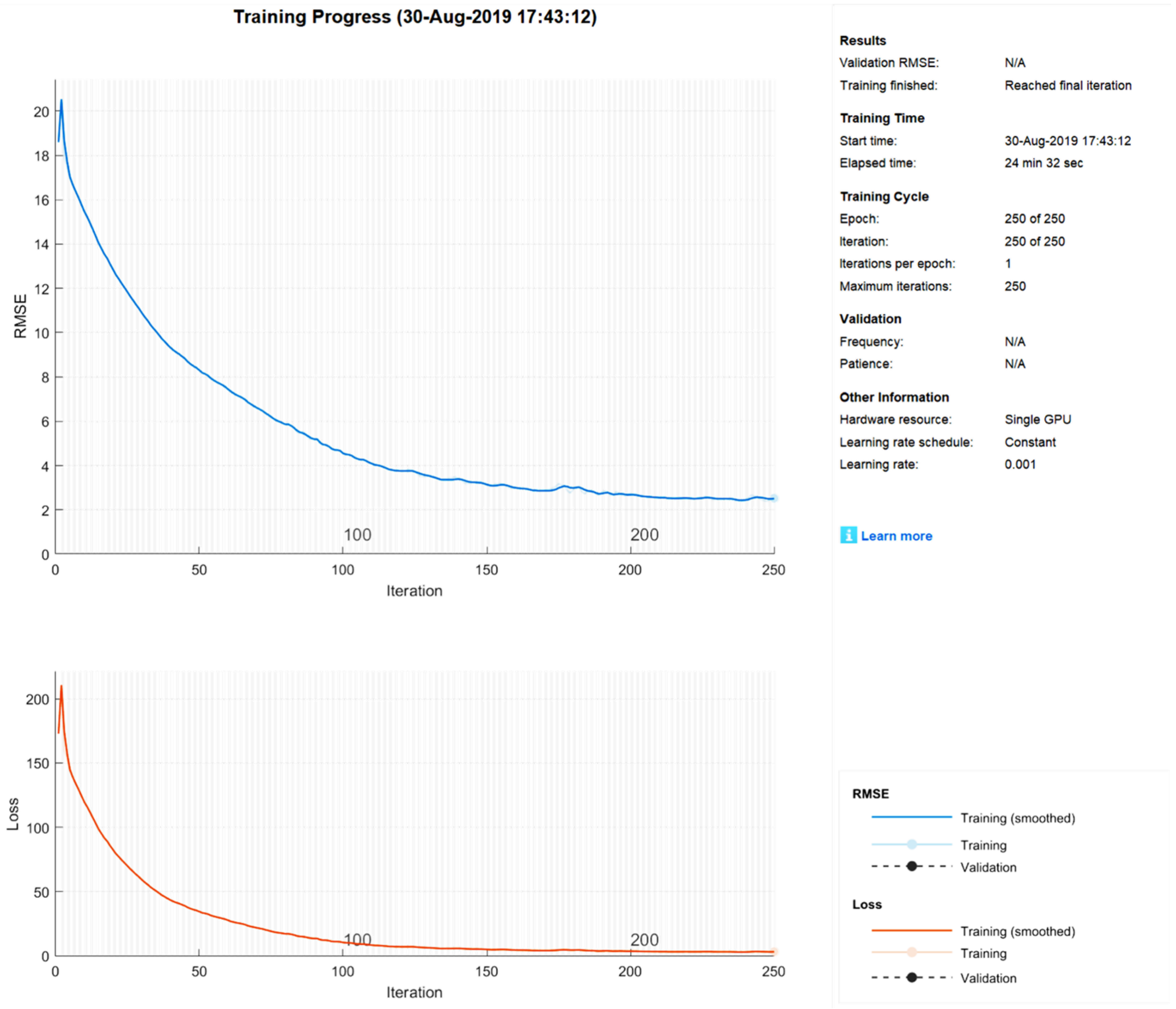

Figure S6: DNN training convergence, measured in the root mean square error (RMSE, top) and the output of the maximum likelihood loss function (bottom). These measures converge to their steady state values within 250 epochs.
